# The Role of Autophagy in the Pathogenesis of Mitochondrial Diseases

**DOI:** 10.3390/cells15151371

**Published:** 2026-07-29

**Authors:** Elena D. Avdonina, Sergey I. Kutsev, Aleksandr V. Shestopalov

**Affiliations:** 1 Federal State Budgetary Scientific Institution Research Centre of Medical Genetic, 1 Moskvorechye St., Moscow 115522, Russia; avdonina.yelena@mail.ru (E.D.A.);; 2 Department of Biochemistry and Molecular Biology, Pirogov Russian National Research Medical University, 1 bldg. 6 Ostrovityanova Ulitsa, Moscow 117513, Russia

**Keywords:** autophagy, selective autophagy, mitophagy, mitochondrial diseases, fatty acid oxidation disorders

## Abstract

**Highlights:**

**What are the main findings?**

- Macroautophagy and its selective forms play an important, multifaceted and often bidirectional role in the pathogenesis of mitochondrial diseases.
- The role of autophagy in cellular organelles, apart from mitophagy, has not been sufficiently investigated.

**What are the implications of the main findings?**

- Restoring autophagy improves mitochondrial function and cell survival in mitochondrial disorders.
- Studying autophagy in cellular organelles, apart from mitochondria, has the potential to reveal new mechanisms underlying the cellular pathogenesis of mitochondrial diseases and to find new promising approaches to treatment.

**Abstract:**

Mitochondrial diseases are a heterogeneous group of inherited disorders caused by defects in the mitochondrial genome or nuclear genes encoding proteins essential for mitochondrial function. These conditions are characterised by progressive dysfunction of tissues with high energy demands, particularly the nervous and muscular systems. In recent years, increasing consideration has been paid to the role of autophagy—the cellular mechanism for the degradation and recycling of intracellular components in the pathogenesis of mitochondrial diseases. This review synthesizes current knowledge on molecular mechanisms of macroautophagy and selective forms of au-tophagy targeting specific organelles and structures: mitophagy, pexophagy, ribophagy, ER-phagy, aggrephagy, lipophagy, lisophagy, and nucleophagy. Using classic mitochondrial syndromes—Kearns–Sayre syndrome (KSS), MERRF, and MELAS, as well as various mitochondrial myopathies—as examples, we discuss experimental evidence indicating both compensatory activation of autophagy and its insufficiency or blockade at different stages. Furthermore, we examine the link between deficiencies of key fatty acid β-oxidation enzymes (VLCAD, MCAD, CPT2) and impaired autophagic flux, including secondary defects of mitophagy mediated by energy deficiency. The review systematises current understanding of how dysregulation of selective autophagy promotes the accumulation of damaged mitochondria, oxidative stress, inflammation, and cell death in mitochondrial diseases. Prospects for therapeutic modulation of autophagy as a potential approach to treating these disorders are discussed.

## 1. Introduction

Mitochondrial disorders are a heterogeneous group of conditions caused by mutations in the mitochondrial or nuclear genome, leading to various impairments in cellular energy metabolism. Primary mitochondrial diseases are caused by mutations in the genes encoding nuclear or mitochondrial proteins involved in oxidative phosphorylation. Examples include Kerns–Sayre syndrome, mitochondrial encephalomyopathy with lactic acidosis and stroke-like episodes (MELAS syndrome), Leber’s hereditary optic neuropathy (LHON), mitochondrial encephalopathy, lactic acidosis, and myoclonic epilepsy with ragged red fibres (MERRF). Secondary mitochondrial diseases arise due to mutations in genes not associated with oxidative phosphorylation (deficiencies in mitochondrial fatty acid oxidation enzymes) or are acquired due to adverse environmental influences (inflammatory response, mitotoxic drugs) [1].

Regardless of the underlying cause, the key link in the pathogenesis of mitochondrial diseases is chronic energy deficiency and oxidative stress caused by Reactive Oxygen Species (ROS). These defects are particularly severe when defective mitochondria are located in muscles, the liver and nervous tissue—tissues that require significant amounts of energy to maintain normal function. At present, there are no effective treatments for mitochondrial diseases, and therapy is aimed at relieving individual clinical symptoms and slowing the progression of the disease [2]. Recent studies have shown that autophagy and mitophagy may play a significant role in suppressing the development of pathological processes associated with mitochondrial dysfunction.

Autophagy is a catabolic process in which cells recycle their own contents to maintain intracellular homeostasis [3]. Through the mechanism of autophagy, the cell adapts to changing environmental conditions and safely disposes of unnecessary or [2] dysfunctional components without triggering apoptosis.

Although the mechanisms have been studied in detail, the role of autophagy in the pathogenesis of mitochondrial diseases remains not fully understood. On the one hand, the selective elimination of damaged mitochondria (mitophagy) is critical for reducing ROS production and preventing the initiation of apoptosis and cell death. On the other hand, the autophagy process itself is energy-dependent, and chronic mitochondrial dysfunction can lead to a reduction in the autophagy flux and the accumulation of toxic substrates.

The role of selective autophagy of other organelles and individual macromolecules in mitochondrial diseases has not been sufficiently studied. Mitochondria do not typically function in isolation but form contacts with other organelles, such as peroxisomes [4], lysosomes [5], and the endoplasmic reticulum [6]. Such a morphofunctional interrelationship may influence the autophagy process under pathological conditions [7,8]. Therefore, a modern approach to the study of mitochondrial pathologies should include consideration of different types of autophagy as an interconnected set of cellular processes directed at maintaining cellular homeostasis under chronic energy deficiency.

The aim of this review is to summarise current evidence on the role of autophagy and selective autophagy in the pathogenesis of mitochondrial diseases, to describe the molecular mechanisms regulating these processes and their interactions, and to evaluate the diagnostic potential of key autophagy markers.

## 2. The Molecular Mechanisms of Autophagy and Selective Autophagy

**Figure 1:**
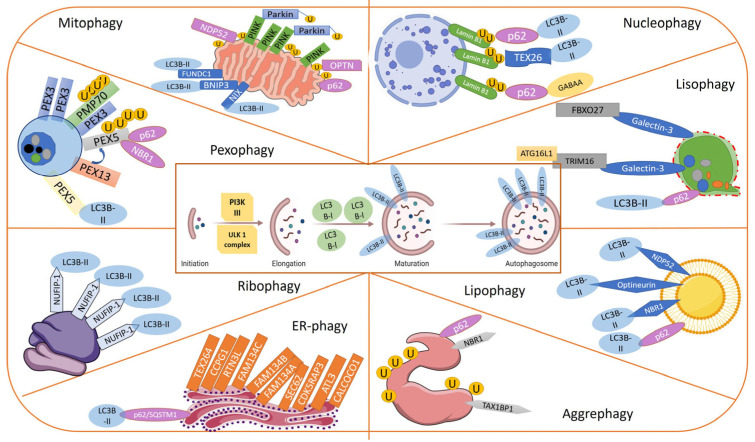
Types of selective autophagy and key receptors and molecules involved in the process. The figure in the centre illustrates the general pathway of the autophagy process. BioArt NIAID Visual & Medical Arts. NIAID NIH BioArt Source. https://bioart.niaid.nih.gov/discover?sort=date_desc&collection=1 (accessed on 4 May 2026).

Autophagy (macroautophagy) is a dynamic process regulated by multiple proteins and protein complexes. The expression of genes associated with autophagy has spatial and temporal specificity. The regulation of autophagic proteins occurs at various levels (transcription, post-transcription, translation and post-translation), which helps to inhibit or activate autophagy at specific stages [9]. During autophagy, various cellular organelles—such as mitochondria, peroxisomes, ribosomes, the endoplasmic reticulum, the nucleus and lysosomes—as well as various substrates—such as proteins and lipids– can be recycled (Figure 1).

The canonical autophagy pathway comprises several stages: initiation (formation of the phagophore), elongation, formation of the autophagosome (a double-membraned vesicle), fusion with a lysosome (formation of an autolysosome), and degradation of the contents. The initiation stage of autophagy begins with mTORC1 (mechanistic target of rapamycin complex 1)—one of the central sensors of energy and nutrients in the cell [10]. Decreased action of growth factors, nutrient depletion, signals from other sensor proteins (increased activity of AMPK, 5′-prime-AMP-activated protein kinase) or cellular stress inhibit mTORC1, triggering autophagy [11]. Inhibition of mTORC1 leads to the dephosphorylation of ULK1 (Unc-51-Like Autophagy Activating Kinase). Following dissociation from mTOR, ULK1 is dephosphorylated, forming the ULK1 complex [12]. Activation of the ULK1 complex stimulates the initiation of membrane detachment from the endoplasmic reticulum to form a phagophore [3]. The ULK complex also phosphorylates the proteins Beclin-1 (BECN1) and vacuolar sorting protein 34 (VPS34), which constitute the catalytic core of the phosphatidylinositol 3-kinase complex (PI3K3). Beclin-1 integrates various cellular signals, including inflammation and immune response signals, and acts as a key crossroads between apoptosis and autophagy. The interaction of the Beclin-1 protein, through the BH3 domain, with anti-apoptotic proteins of the Bcl-2 family inhibits autophagy whilst increasing the threshold for apoptosis [13]. The maintenance of this balance by Beclin-1 is crucial for cell survival. Beclin-1 is also involved in the functioning of exogenous signalling pathways mediated by death receptors. Cleavage of Beclin-1 by caspases (activated through death receptors) results in the N-terminal fragments of Beclin-1 being relocalised to the nucleus or mitochondria, enhancing the cellular apoptotic response [14].

During the elongation phase, the PI3K-III complex generates phosphatidylinositol 3-phosphate (PI3P) on the phagophore membrane, which facilitates the recruitment of the ATG12–ATG5–ATG16L1 complex. It functions as an E3 ligase for the lipidation of proteins in the ATG8 family, in particular LC3B (microtubule-associated protein 1A/1B light chain 3). As a result, the cytosolic protein LC3B-I becomes covalently linked to phosphatidylethanolamine in the membrane, converting into the lipid-modified form LC3B-II, which is incorporated into the phagophore membrane and participates in the formation of the autophagosome [15]. Finally, the autophagosome membrane fuses with the lysosomal membrane, which carries lysosome-associated membrane glycoprotein-1 (LAMP1) on its surface and contains hydrolytic enzymes. This results in the formation of a mature autophagosome, where the cargo is degraded [16].

Mitochondrial autophagy, also known as mitophagy, is an important process in sustaining mitochondrial and cellular homeostasis [17]. In the case of nutrient deficiency, the action of ROS or the accumulation of mutations in mitochondria, depolarisation and loss of membrane potential occur. This triggers the autophagy mechanism for the selective clearance of damaged or dysfunctional mitochondria. Currently, two categories of mitophagy mechanisms are identified: ubiquitin (Ub)-dependent and Ub-independent pathways.

Ubiquitin-dependent pathways of mitophagy activation: Among these mechanisms, the pathway involving PTEN-induced kinase 1 (PINK1) and Parkin is the most extensively studied. In healthy mitochondria, the level of the mitochondrial serine/threonine kinase PINK1 on the outer mitochondrial membrane is very low; however, upon irreversible mitochondrial damage, PINK1 accumulates on the outer membrane and recruits the E3 ubiquitin ligase Parkin [18]. Parkin ubiquitinates proteins on the mitochondrial surface, creating signalling chains that are recognised by mitophagy receptors (OPTN, NDP52, p62), leading to the enveloping of the mitochondria by an autophagosome and its subsequent degradation: Parkin also ubiquitinates mitofusin 2 (Mfn2), causing its degradation and thereby preventing the fusion of the damaged mitochondrion with the healthy network, which contributes to the isolation of the mutant organelle [19]. Ubiquitin-independent pathways of mitophagy activation: The outer mitochondrial membrane contains proteins that act as autophagy receptors. These can bind directly to LC3B without ubiquitination, thereby inducing mitophagy. In mammals, these receptors include a protein similar to BCL2-interacting protein 3 (BNIP3L/NIX), BCL2-interacting protein 3 (BNIP3), FUNDC1 (a protein containing the FUN14 domain) and others.

Pexophagy is a selective form of autophagy that targets damaged or excess peroxisomes—specialised single-membrane organelles involved in fatty acid oxidation, the synthesis of esterified lipids, and the regulation of redox homeostasis [20]. Pexophagy can be induced by an excess of peroxisomes, disruption of peroxisomal protein import, nutrient depletion, hypoxia and ROS. Most of these stimuli promote the ubiquitination of peroxisomal membrane proteins (PMPs), which activate autophagy receptors and form a bridge between damaged peroxisomes and the growing phagophore for their degradation in autolysosomes.

Some peroxisomal proteins involved in the activation of peroxisomal autophagy:

- PEX3 is required for the incorporation of PMPs into the membrane of newly forming peroxisomes; increased levels of PEX3 promote their ubiquitination. Inhibition of the autophagy receptor neighbour of BRCA1 gene 1 (NBR1) reduces PEX3-induced autophagic degradation of peroxisomes.
- PMP70: During starvation and upon inhibition of mTORC1, the activity of the E3 ubiquitin ligase (or MARCHF5) is reduced, leading to the ubiquitination of PMP70 and the induction of pexophagy [21]. Deubiquitination of PMP70 by ubiquitin-specific peptidase 30 (USP30), a mitochondrial deubiquitinating enzyme, leads to the inhibition of pexophagy.
- PEX5 promotes the import of PMPs into peroxisomes. Ubiquitination of PEX5 in response to ROS accumulation, hypoxia and mTORC1 inhibition activates p62 and NBR1 to trigger peroxisomal autophagy.
- PEX13 is responsible for the import of peroxisomal matrix proteins; disruption leads to the accumulation of ubiquitinated PEX5 and peroxisome-dependent ROS, which induce pexophagy [22].
- During starvation, PEX14 binds directly to the lipid-bound form of LC3B (LC3B-II), thereby enhancing the fusion of the peroxisome with the autophagosome [23].

Ribophagy is a process involving the specific degradation of ribosomes [24]. In mammals, ribophagy is activated under starvation conditions through the inactivation of mTORC1, with the involvement of a specific receptor on the ribosomes—nuclear fragile X protein 1 (NUFIP-1). NUFIP-1 can bind to the large 60S ribosomal subunit through a protein containing a zinc finger domain (ZNHIT3) and directly to the LC-3B protein [25]. The specific mechanisms of ribophagy require further study.

Reticulophagy (ER-phagy) is the selective elimination of damaged or excess endoplasmic reticulum (ER) membranes via the autophagy system. The endoplasmic reticulum (ER) is the largest organelle in the cell and is involved in processes such as protein secretion, lipid synthesis, calcium accumulation and organelle biogenesis. Therefore, the regulation of its function via ER-phagy is significant for the cell. ER-phagy can occur either via the involvement of autophagosomes (macro-ER-phagy) or through direct fusion with lysosomes (micro-ER-phagy) [26].

Eleven ER-phagocytic receptors have been identified in mammals: FAM134B, FAM134A, FAM134C, RTN3L, CCPG1, SEC62, TEX264, ATL3, CALCOCO1, p62 and CDK5RAP3. These receptors varied in their molecular characteristics and functions, but shared similar LC3B-interacting regions (LIRs) or Atg8-interacting motifs (AIMs) for interacting with proteins of the ATG8 family, and induced the degradation of various ER regions under different physiological or pathological conditions [27]. Particular note should be made of the role of the most extensively studied protein, p62/SQSTM1, which facilitates the removal of ubiquitinated components—defective or excess ER membranes—thereby ensuring ER quality control. It functions as an autophagy receptor and adaptor, linking damaged components to autophagosomes via interaction with LC3B [28].

An alternative mechanism in mammals may involve interaction between ER-phagy receptors and components of the autophagy initiation complex, such as FIP200, ULK1 and ATG13 [26].

Lysophagy is a form of selective autophagy in which the cell isolates and digests lysosomes. Lysosomes are organelles that play a key role in the autophagy process by breaking down macromolecules and organelles, thereby maintaining cellular homeostasis. As lysosomes are the major degradative organelles, they are at increased risk of damage. Depending on the extent of the damage, cells activate various mechanisms to maintain lysosomal integrity. Lysophagy is activated when lysosomes cannot be restored due to an irreversible increase in their permeability [29].

Damage to the lysosomal membrane leads to the exposure of intraluminal glycans, which act as damage signals recognised by cytosolic galectin-3 and the ubiquitination system. Galectin-3 also inhibits mTOR activity, leading to the translocation of TFEB into the nucleus, which promotes lysosome biogenesis [29]. The E3 ubiquitin ligases TRIM16 and FBXO27 initiate ubiquitination, and TRIM16 acts as a link between damaged lysosomes and ATG proteins, facilitating the formation of the autophagosome membrane. At the same time, the endolysosomal damage response (ELDR) complex in lysosomes leads to the recruitment of LC3B to initiate autophagosome formation. Then, the fusion of autophagosomes with functional lysosomes leads to the degradation of damaged lysosomes [30].

Nucleophagy is a selective form of autophagy that specifically targets nuclear components or structures: nuclear proteins, micronuclei, fragmented chromosomes and DNA-protein cross-links. The autophagic receptors p62 and Testis Expressed 264 (TEX264) recognise nuclear cargo and bind to LC3B or GABAA receptor-associated protein (GABARAP), which are located on the phagophore membranes. This nuclear cargo is then delivered to lysosomes for degradation [31].

Lipophagy is a form of autophagy in which lipid droplets—the primary lipid-storing organelles of eukaryotic cells—are degraded. Lipid droplets (LDs) are now being studied not merely as stores of triacylglycerides, but also as dynamic organelles that are continuously formed, altered, and transported, and that participate in interactions with other compartments in lipid metabolism, signalling regulation, and proteostasis [32]. Lipophagy is regulated by systems that recognise the cell’s nutritional status, such as the nuclear receptors farnesoid X receptor (FXR) and peroxisome proliferator-activated receptor alpha (PPARα), cAMP response element-binding protein (CREB), mTOR and AMPK [33]. Lipophagy can also be activated as a protective mechanism against lipotoxicity.

Lipophagy initiates with the recognition of the cargo by the autophagosome membrane through interaction with the LC3B protein. Generally, this process involves adaptor proteins such as p62, Optineurin, Neighbour of BRCA1 gene 1 protein (NBR1) and Nuclear Domain 10 Protein 52 (NDP52), which bridge the organelle membrane to the LC3B protein. The Huntingtin protein is essential for the activation of lipophagy under stress conditions, linking p62 to LC3B-II, as well as activating the pro-autophagic kinase ULK-1 [34]. Proteins of the Rab family, such as Rab7, Rab10 and Rab25, may also play a role in the regulation of lipophagy [35]. After being engulfed by lysosomes, the lipid cargo is digested by specific lysosomal acid lipases, which are capable of functioning in an acidic (pH = 4.5–5) environment and catabolise triacylglycerides, diacylglycerides, cholesterol esters and retinol esters [35].

Aggrephagy is a form of autophagy that degrades misfolded or aggregated proteins, which can accumulate within the cell and disrupt its functions. To degrade these proteins, the ubiquitin–proteasome pathway is primarily activated, enabling the cleavage of misfolded proteins tagged with Ub. When the proteasome cannot process the increasing number of Ub aggregates, the autophagy process is activated, involving the receptors p62, NBR1 and Tax1 Binding Protein 1 (TAX1BP1). These receptors induce the formation of an autophagosome, which engulfs the protein aggregates and fuses with a lysosome for their digestion [36].

Recent studies have also revealed that the Chaperonin Containing TCP-1 (CCT2) protein subunit may act as an autophagy receptor during protein aggregation via a unique mechanism. CCT2 interacts with proteins prone to aggregation, regardless of their ubiquitination status [37]. CCT2-mediated aggrephagy occurs independently of canonical autophagy receptors, including p62, NBR1 and TAX1BP1 [38].

## 3. Dysfunction of Autophagy and Organelle-Specific Autophagy as a Potential Pathogenic Mechanism in Mitochondrial Diseases

**Figure 2:**
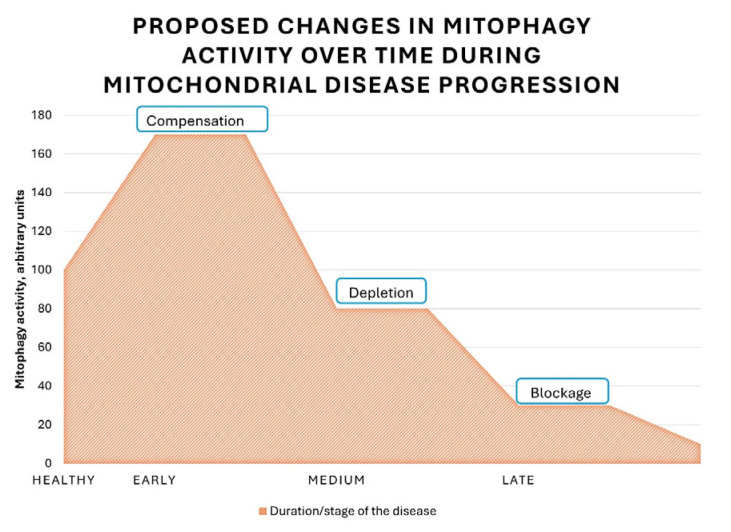
Graph of proposed changes in mitophagy activity over time during mitochondrial disease progression. The horizontal axis shows the duration/severity of the disease (from early to late stage). The vertical axis shows mitophagy activity in arbitrary units. The curve initially rises above normal (compensatory activation), then drops sharply below normal (exhaustion/block).

Mitochondrial dysfunction in mitochondrial disorders can be identified by increased ROS levels, membrane depolarisation, damage to mtDNA, cristae disorganisation and reduced ATP synthesis [16]. Tissues with the highest metabolic activity, such as skeletal and cardiac muscles, as well as the brain, eyes and inner ear, are the first to be damaged. In mitochondrial diseases, the activity of autophagy [39] and, in particular, mitophagy [40], is not static and can vary significantly—from compensatory enhancement in the early stages to depletion and blockage in the later stages, which exacerbates the course of the disease (Figure 2). The success of therapy for mitochondrial diseases may potentially depend on the ability to maintain an adequate level of mitophagy, preventing its depletion and the triggering of apoptosis.

Most research on selective autophagy in mitochondrial dysfunction has focused on mitophagy, whereas data on the autophagy of other organelles are limited. Nevertheless, a comprehensive study of these processes in mitochondrial diseases is a promising direction that may help identify new therapeutic approaches for correcting mitochondrial disorders.

In recent years, an increasing number of studies have focused on the role of autophagy in the pathogenesis of mitochondrial diseases. Here are some examples of mitochondrial disorders, focusing on how organelle-specific autophagy affects the course of the disease. We will also review progress in the search for specific molecules capable of modulating these processes to relieve clinical symptoms.

### 3.1. Classic Mitochondrial Syndromes

**Kerns–Seyer syndrome (KSS)** is a rare multisystemic mitochondrial disorder caused by changes in mitochondrial DNA (mtDNA), primarily deletions ranging from 1.1 to 10 kb (most commonly Δ4977 kb).

The exact mechanisms of inheritance for this condition have not been fully understood. KSS is a more severe syndromic variant of chronic progressive external ophthalmoplegia (CPEO). This syndrome is characterised by isolated injury to the eyelid and eye muscles, leading to ptosis and ophthalmoplegia, respectively; bilateral pigmentary retinopathy; and heart block. Additional features may include cerebellar ataxia, muscle weakness, deafness, diabetes mellitus, growth hormone deficiency, hypoparathyroidism or other endocrine disorders [41].

A comprehensive study conducted in 2007 proposed a mechanistic hypothesis about the consequences of mtDNA deletions in patients with KSS and CPEO [42]. The authors investigated the global expression profile of nuclear genes in muscle biopsies, myoblasts, fibroblasts and lymphoblasts in controls and patients with CPEO or KSS. It was shown that key tRNAs and functional components of the respiratory chain in patients with mutations in the relevant genes exacerbated the misfolding and damage of mitochondrial proteins. Gene induction was also observed in genes involved in the protein stress response: PRKR-Like Endoplasmic Reticulum Kinase (PERK), Heat Shock Protein Family A (HSPA4) and DnaJ Heat Shock Protein Family (DNAJC3, DNAJC4), as well as significant levels of oxidised proteins, which may have inhibited the activity of the ubiquitin–proteasome system. This leads to the depletion of amino acids in the cell, which reduces mTOR activity and subsequently activates autophagy [16]. Furthermore, during the development of degenerative processes, including KSS, there is often an increase in ROS generation, leading to the activation of autophagy via the activation of the AMPK signalling cascade and the ULK1 complex, disruption of the interaction between Beclin-1 and Bcl-2, alteration of mitochondrial homeostasis and membrane depolarisation [42].

A recently published study [43] highlights the fact that insufficient attention has been paid in the literature to processes such as autophagy and apoptosis in KSS. The author suggests that research into autophagy in KSS will be useful for a better understanding of the underlying mechanisms of the disease and may also lead to the development of new treatment strategies.

At present, no therapeutic agents have been identified that can alleviate the condition in KSS. As a form of replacement therapy for KSS, some studies suggest incorporating free amino acid supplements into patients’ diets to stimulate mTOR and prevent the activation of autophagy [42].

**MERRF syndrome (myoclonic epilepsy with ragged-red fibres)** is a rare mitochondrial disorder belonging to the group of hereditary mitochondrial encephalomyopathies.

It is caused by mutations in the mitochondrial gene Mitochondrially Encoded tRNA-Lys (MTTK), which encodes lysine-carrying tRNA—responsible for more than 90% of all mutations—or in the genes MTTF (phenylalanine-carrying tRNA), MTTH (tRNA carrying histidine), MTTI (tRNA carrying isoleucine), MTTL1 (tRNA carrying leucine), MTTP (tRNA carrying proline), MTTS1 and MTTS2 (tRNA carrying serine), which represent less than 5%.

The most common mutation is a single nucleotide substitution at position 8344 (m.8344A>G) in the MTTK gene (>80% of mutations). This amino acid is a component of mitochondrial proteins essential for the normal functioning of the respiratory chain. Therefore, mutations in this gene cause an energy deficit in tissues, especially in muscle and nerve tissue. This leads to the clinical symptoms of MERRF syndrome: myoclonus, myoclonic epilepsy, cerebellar ataxia, and muscle weakness [44].

The role of mitophagy as a cell survival mechanism in MERRF syndrome is a key area of research. The authors of a single study on the role of autophagy in the progression of MERRF syndrome [45] explored the pathophysiology of the m.8344A>G mutation in primary cultured skin fibroblasts derived from patients with MERRF (73% heterozygosity) and cybrids with 100% heterozygosity. In both MERRF cell models, increased expression of autophagic proteins, mediated by AMPK, and enhanced activation of mitophagy, mediated by Parkin, were observed. This demonstrates that the activation of mitophagy is a mechanism for cell survival.

This study also describes a reduction in the levels of autophagic proteins in MERRF fibroblasts and cybrids upon the addition of coenzyme Q10. It is suggested that the addition of coenzyme Q10 may prevent the translocation of Parkin into the mitochondria and stimulate autophagy and mitophagy, thereby partially improving cellular pathophysiology. However, this effect is controversial and requires further research [45]. No specific therapy for MERRF syndrome has yet been developed.

**Mitochondrial encephalomyopathy with lactic acidosis and stroke-like episodes (MELAS)** is a rare inherited mitochondrial disorder that mostly affects the nervous system and muscles. It manifests as recurrent episodes of encephalopathy, myopathy, headache and focal neurological deficits, usually between the ages of 2 and 15. A distinctive feature of the syndrome is the occurrence of stroke-like episodes leading to hemiparesis, hemianopia or cortical blindness [46].

The cause of this syndrome (similar to MERRF) is a nucleotide substitution in tRNA. Currently, at least 23 single-nucleotide mutations and one deletion are known in the MTTL1, MTTH, MTTK, MTTS1, MTTS2, and MTTQ (glutamine-carrying tRNA) genes, as well as in the Mitochondrially Encoded NADH Dehydrogenase (MTND1, MTND5, MTND6) genes [47].

The most prevalent mutation is in the mitochondrial MTTL1 gene. The single-nucleotide variant m.3243A>G is found in 80% of affected patients, whilst the second most common variant, m.3271T>C, is observed in 10%. A distinctive feature of MELAS syndrome is the accumulation of damaged mitochondria in muscle fibres, the number of which increases in an attempt to compensate for the energy deficiency. Using Gomori’s trichrome stain, they appear bright red in contrast to the blue myofibrils, a feature that came to be known as «ragged red fibres» [46].

There are a series of studies on MELAS that examine in detail the role of mitophagy and the general autophagic pathway, sometimes with contradictory conclusions.

In the study by Bhattacharya S. et al. (2022) [48], induced pluripotent stem cells (iPSCs) derived from skin biopsies of MELAS patients (m.3243A>G tRNA Leu) with varying levels of mtDNA heteroplasmy were differentiated into retinal pigment epithelium (RPE). MELAS-RPE cells exhibited reduced oxidative phosphorylation and increased glycolysis, which simulated the energy deficit in MELAS syndrome. A reduction in AMPKα activity was shown in MELAS-RPE cells, presumably associated with impaired mitochondrial homeostasis. Increased activation of signal transducer and activator of transcription 3 (STAT3) was also observed, which primarily disrupts the formation of the BECN1/PIK3C3 complex [49], leading to inhibition of the autophagic flux, the extent of which depended on the percentage of heteroplasmy. In addition, lysosomal dysfunction (decreased LC3B-II degradation caused by cathepsin D dysfunction in lysosomes) and disrupted mitochondrial recycling (increased PINK1 synthesis on damaged mitochondria and their accumulation) also contributed to the autophagy defect [48].

The authors of another study [50] investigating iPSCs from patients with MELAS report an increase in the autophagic flux, with a clear increase in ROS production, intracellular calcium accumulation, and sustained depolarisation of the mitochondrial membrane potential under energy-deprived conditions. Increased levels of LC3B-II, a marker of autophagosome and autolysosome accumulation, were also detected. Notably, Parkin levels did not differ from those in the control group of healthy cells, which, as the authors suggest, indicates the activation of mitophagy via a Parkin-independent pathway.

Another group of authors [51] worked on RNA sequencing, electron microscopy and biochemical analyses of skeletal muscle or fibroblasts obtained from patients with MELAS. The data did not show any changes at the transcriptional level in genes or autophagy pathways in the skeletal muscle of patients with MELAS. However, it is interesting to note that functional tests indicated mitochondrial dysfunction (significantly reduced levels of Beclin-1 and LC3B-II proteins) and a deficiency in degradation due to reduced lysosome content.

There is currently no specific, standardised approach to the treatment of MELAS syndrome. Therapy is focused on alleviating symptoms, for example, through the supplementation of L-arginine, L-carnitine or coenzyme Q10. However, the efficacy of such treatment remains questionable [52].

Finsterer J. and Barton P. (2010) proposed the antiepileptic drug lamotrigine for the treatment of patients with MELAS, as it helped to control seizures and prevent stroke-like episodes [53]. Recent studies [54], on the other hand, have proposed nitazoxanide as a candidate drug. This is a precursor form of tizoxanide, a widely used antiprotozoal drug. It has been shown that tizoxanide stimulates the transcription of mitochondrial nuclear retrograde regulator 1 (MNRR1). The authors suggest that activation of MNRR1 enhances mitophagy (via increased expression of PINK1) and mitochondrial biogenesis, which could potentially improve the condition of patients with MELAS.

In an experiment by Chung C.Y.’s group [55], it was shown that in fibroblasts from patients with the m.3243A>G mutation, the PI3K-AKT-mTORC1 axis, which normally suppresses autophagy, was in a state of constitutive activation, leading to a block in the clearance of damaged mitochondria from the cell. Modulation of this pathway by rapamycin (mTORC inhibitor) restored the autophagic flux and reduced the mutant load through the activation of mitophagy. Furthermore, short-term inhibition of the PI3K-AKT-mTORC1 pathway contributed to the restoration of mitochondrial membrane potential and improved oxidative phosphorylation. The authors also report that the compound LY294002 also contributed to the enhancement of autophagy. This compound non-selectively inhibits both class 1 PI3K, which induces autophagy, and class 3 PI3K, which inhibits it. This may have a dual effect on autophagy activation and may be concentration-dependent. This issue requires further research.

### 3.2. Mitochondrial Myopathies with Autophagic Defects

**GNE myopathy (Nonaka myopathy)** is a rare distal myopathy with a late debut (between the ages of 20 and 40) and an autosomal recessive inheritance. It is characterised by weakness of the distal muscles in the legs, with slow progression to the proximal muscles of the lower limbs (thighs) and upper limbs (arm muscles). The neck flexor muscles are also frequently affected [56].

GNE myopathy is caused by biallelic mutations in the GNE gene (9p13.3), which encodes a bifunctional enzyme that initiates and regulates the biosynthesis of N-acetylneuraminic acid, a precursor of sialic acids. Mutations in this gene lead to a 30–60% reduction in enzyme activity, resulting in reduced sialylation of glycoproteins and glycolipids. Hyposialylation likely contributes to the pathogenesis of the disease, but the mechanism by which mutations in the GNE gene lead to muscle disease remains largely unexplored [57].

Dysregulation of autophagy in skeletal muscle is considered a factor in muscle atrophy and dystrophy, leading to a reduction in regenerative capacity due to the depletion of stem cells. Muscle biopsies from patients with GNE myopathy are also characterised by the presence of rimmed vacuoles, surrounded by a large amount of autophagic material and containing tubulo-filamentous aggregates [56].

The role of autophagy in Nonaka myopathy remains largely unexplored. One of the few studies [58] using fibroblasts obtained from patients with GNE myopathy demonstrated abnormal autophagy activity due to increased expression of LC3B-II and p62 proteins around vacuoles in muscle biopsies. The expression levels of Beclin-1, AMBRA, LC3B-II and p62 in GNE myopathy cells were significantly higher than in healthy cells under normal culture conditions. However, under starvation conditions (absence of serum in the medium for 10 h), a decrease in Beclin-1 and AMBRA levels was observed in myopathy-affected cells, whereas the expression of LC3B-II and p62 was increased compared with the control group. These results demonstrate that both autophagy initiation and the autophagic flux are impaired in fibroblasts with GNE myopathy. The addition of metformin to fibroblasts with GNE myopathy under starvation conditions resulted in enhanced autophagy and improved cell viability [58]. It has been shown that metformin treatment inhibits the AMPK–mTOR signalling pathway and, furthermore, exerts a protective effect via mechanisms independent of AMPK–mTOR.

Transcriptome analysis of two independent GNE myoblast models derived from human pluripotent stem cells identified several genes associated with autophagy as pathogenic signatures of GNE myopathy [59]. Transcriptome analysis predicted that abnormal activation of the non-canonical AKT–mTORC1 pathway induces ULK1 phosphorylation, thereby suppressing the initiation of autophagy and increasing the risk of muscle atrophy. In vitro treatment with copanlisib—a PI3K inhibitor—reactivated the ULK1 pathway and normalised autophagy in both a C2C12 mouse myoblast model and an isogenic human pluripotent stem cell-derived neuromuscular organoid model (NMOs). These results demonstrate the link between mTOR hyperactivation and defects in autophagy in GNE myopathy. Copanlisib is a promising therapeutic agent aimed at correcting autophagy.

Hydroxyethylamine-based molecules—LTC-181 and LTC-1717—also had a positive effect on maintaining the viability of cells with GNE myopathy [60]. After treatment of cells with a knockout of exon 3 in the GNE gene, approximately 13% and 80% increases in the LC3BII/I ratio were observed compared with untreated cells, which indicates activation of autophagy.

**Autophagic vacuolar myopathy** is a group of rare genetic disorders characterised by the accumulation of autophagic vacuoles in the myocytes of skeletal and cardiac muscle tissue, leading to myopathy and cardiomyopathy. The condition can manifest at any age—from early childhood to late adulthood.

This group of disorders includes Danon disease—a rare inherited disorder from the group of lysosomal storage diseases, which is X-linked dominant. The main clinical symptoms are cardiomyopathy, damage to skeletal muscle and the retina, and intellectual developmental disorders [61]. Danon disease is caused by a mutation in the LAMP2 gene, which disrupts the fusion of autophagic vacuoles and lysosomes, leading to their accumulation and impaired macroautophagy. Most LAMP2 mutations are assumed to lead to a deficiency of all three isoforms of the gene, and isoform-specific mutations have been recorded only for LAMP2-2b, suggesting that a deficiency of LAMP2-2B is enough to cause the disease [62]. Since LAMP-2B is primarily expressed in cardiac and skeletal muscle and the brain, the functions of these tissues will be the primary ones to be impaired.

Studies show that defects in genes regulating autophagy directly lead to disease. To better understand the molecular mechanisms of Danon disease, a group of authors led by Barndt R.J. et al. (2023) [63] proposed a model using iPSCs with a LAMP2 gene knockout, differentiated into cardiomyocytes and treated with maturation medium to stimulate metabolic and functional maturity. On this model, the authors demonstrated that Danon disease causes disruption of the autophagic flux, sarcomere dislocation, and dysregulation of calcium circulation in cardiomyocytes. The study also notes in particular that disruption of the autophagy flux can lead to the accumulation of dysfunctional mitochondria and an increase in cytosolic and mitochondrial ROS, which could potentially activate the process of mitophagy.

An earlier study [64] also reported that mitochondrial dysfunction plays a significant role in the development of Danon disease. Impaired mitochondrial metabolism, oxidative stress and energy deficiency exacerbate the progression of the disease and may be a central factor in its pathogenesis and a potential therapeutic target. In a study by Xu Z. et al. (2018) [65] using a mouse model with LAMP2 deficiency, the lipidomic profiles of liver, plasma and retinal tissues were described for the first time. The authors showed that LAMP2 knockout mice had higher levels of free fatty acids in the liver and retina and lower levels in plasma, whilst levels of triglycerides, phosphatidylcholine and phosphatidylethanolamine were reduced in all tissues. This implies a significant role for LAMP2 in autophagy, the maintenance of energy balance and metabolic stability, and a possible role of lipophagy in the development and manifestation of Danon disease.

The development of specific therapy for Danon disease generally involves correcting the LAMP2 mutation through gene therapy, using such tools as adenovirus-associated viral vectors [66].

On the other hand, research into traditional drugs for the treatment of heart failure and ventricular remodelling, such as Ramipril [67], is continuing.

An area of research is developing focused on the creation of animal and cellular models with the Danon disease phenotype and the identification of targets for the activation of autophagy. Dvornikov A.V. et al. (2019) [68] demonstrated in a Danio rerio model with the Danon disease phenotype that mTOR inhibition reduces the adverse effects of impaired autophagy and partially mitigates the primary metabolic dysfunctions.

**Myofibrillar myopathy** is a group of inherited disorders characterised by damage to the cardiac and skeletal muscle tissue. Myofibrillar myopathy is caused by mutations in the genes encoding muscle proteins: desmin (DES), α-B-crystallin (CRYAB), myotilin (MYOT), LIM-domain-binding protein 3 (LDB3), filamin C (FLNC), BCL2-associated atanogen 3 (BAG3) and others. Mutations in the MYOT, LDB3, FLNC and BAG3 genes are inherited in an autosomal dominant way, mutations in the CRYAB gene are inherited in an autosomal recessive way, and mutations in the DES gene can be transmitted in both ways [69]. The clinical pattern of myofibrillar myopathy is highly variable. A characteristic feature of myofibrillar myopathy is the degradation of Z-discs in muscle fibres inside sarcomeres, leading to the aggregation of proteins in the muscles, disruption of the connection between sarcomeres, and a reduction in myofibrillar strength [70].

Several studies [71,72] demonstrate the key role of aggrephagy in all genetic forms of MFM. A significant increase in the aggrephagy markers p62/SQSTM1 (an autophagic receptor capable of binding to polyubiquitinated proteins and LC3B), NBR1 (a selective autophagic receptor also capable of binding to LC3B) and FK2 (a ubiquitinated protein) compared with the control group indicates the activation of this pathway as an attempt by the cell to cope with the accumulation of toxic proteins. The highest activation of aggrephagy was observed in patients with a mutation in the MYOT gene.

The link between MFM and other forms of selective autophagy remains unclear, although some studies have provided a number of important observations. A fundamental study [73] in mice with MFM (a mutation in the BAG3 gene) has shown that, alongside defects in protein synthesis, the inhibition of macroautophagy and impaired mitophagy play a key role in the pathogenesis of the disease. Recovery of autophagy by in vivo gene therapy partially restored muscle function, which proves its central role. Since BAG3 is part of the chaperone-assisted selective autophagy (CASA) complex, which is involved in the degradation of proteins damaged by physical stress, its dysfunction may indirectly affect reticulophagy processes.

No specific therapy for MFM has yet been developed. Research is currently underway [74] into the use of metformin to remove protein aggregates from muscles by activating autophagy.

### 3.3. Diseases Caused by a Deficiency of Mitochondrial Fatty Acid Oxidation Enzymes

Constitute a group of inherited disorders resulting from a deficiency of the enzymes involved in mitochondrial β-oxidation of fatty acids (β-FAO). Mutations in these enzymes lead to impaired fatty acid metabolism in the mitochondria, accompanied by clinical symptoms such as acute hypoketotic hypoglycaemia, hepatomegaly and steatosis, hypertrophic cardiomyopathy, and skeletal muscle atrophy. In mild cases, the condition leads to an inability to tolerate fasting and intense physical activity, whilst in severe cases it leads to coma or death [75].

There are a large number of different genetic disorders in humans in which fatty acid oxidation in the mitochondria is disrupted. Recessively inherited disorders have been identified for almost all genes encoding fatty acid oxidation enzymes and transporters [76]. Below, we will review examples of such diseases, focusing on the role of autophagy in them.

**Very-long-chain acyl-CoA dehydrogenase (VLCAD)** deficiency is an autosomal recessive disorder caused by mutations in the ACADVL gene, located on the short arm of chromosome 17, which encodes the enzyme very-long-chain acyl-CoA dehydrogenase. The mitochondrial enzyme VLCAD is located on the inner mitochondrial membrane and catalyses the initial stage of mitochondrial β-FAO for fatty acids with a chain length of 14 to 20 carbon atoms. Fatty acids with a chain length of more than 20 carbon atoms first undergo several stages of oxidation in peroxisomes [77].

In the event of reduced VLCAD expression, long-chain fatty acids cannot be metabolised, which leads to metabolic distress due to chronic energy deficiency. Furthermore, the accumulation of long-chain fatty acids and their metabolites is toxic and causes damage to cells and tissues [78]. This primarily affects the function of cardiac tissue (arrhythmias, hypertrophic cardiomyopathy), hepatic tissue (fatty infiltration, panlobular steatosis) and muscle tissue (hypotonia, myoglobinuria, increased creatine kinase activity) [79].

**Medium-chain acyl-CoA dehydrogenase (MCADD)** deficiency is the most common form of β-FAO disorder, caused by a mutation in the ACADM gene on the short arm of chromosome 1, which encodes the mitochondrial enzyme medium-chain acyl-CoA dehydrogenase (MCAD). The flavin-containing enzyme MCAD is located in the mitochondrial matrix and is involved in the β-oxidation of fatty acids with carbon chain lengths of C4–C12 [80]. The clinical manifestations of MCAD deficiency are similar to those seen in VLCAD deficiency.

**Carnitine palmitoyltransferase deficiency (CPT1, CPT2)** is a rare autosomal recessive inherited disorder belonging to the group of fatty acid transport disorders. The condition is caused by mutations in the CPT1A and CPT2 genes. The enzyme carnitine palmitoyltransferase (CPT) transports long-chain fatty acids from the cytoplasm into the mitochondria for oxidation. CPT exists in two subforms—CPT I (located on the outer mitochondrial membrane) and CPT II (located inside the mitochondria). Together with carnitine–acylcarnitine translocase (CACT), these enzymes play a critical role in the transport of fatty acids across the mitochondrial membrane [81]. Mutations in these genes reduce enzyme activity, which decreases the rate at which acylcarnitines enter the cell and causes an energy deficit.

CPT I deficiency is clinically characterised by acute metabolic episodes triggered by starvation, fever or infection, hepatic encephalopathy and hypoglycaemia. In severe cases, metabolic decompensation can lead to neurological damage in the form of psychomotor disturbances and cognitive impairment. Outside of these episodes, the person appears healthy [82]. CPT II deficiency manifests as a wide range of symptoms, which depend on the age of onset and the severity of the condition: from episodes of myalgia, weakness and rhabdomyolysis to liver failure, hypoketotic hypoglycaemia, cardiomyopathy, respiratory failure and/or cardiac arrhythmia [83].

**Figure 3:**
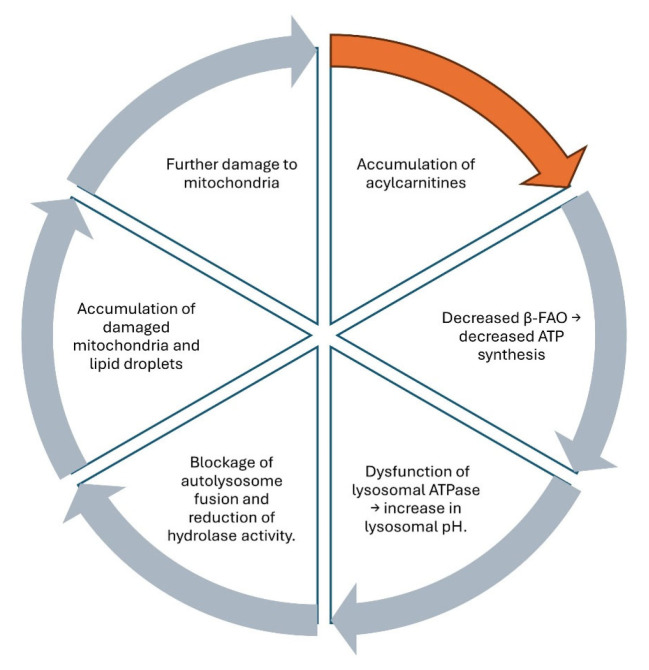
The “vicious cycle” of autophagy in fatty acid oxidation deficiencies (VLCAD/MCAD/CPT2 deficiency). The orange arrow indicates the conditional onset of autophagy impairment in fatty acid oxidation deficiencies.

The autophagy pathway in cases of VLCAD, MCAD and CPT2 enzyme deficiencies is also characterised by the formation of a vicious circle (Figure 3). The trigger is the accumulation of acylcarnitines within the cell, which cannot be utilised in β-FAO. This increases the burden on autophagy processes against a growing energy deficiency, leading to a further increase in acylcarnitines in the cell and exacerbating the situation.

Recent studies have shown that deficiencies in key fatty acid β-oxidation enzymes (such as VLCAD and CPT2) not only impair energy metabolism but also disrupt autophagy mechanisms. According to a study by Angelini C. et al. (2016) [84], based on muscle biopsies from patients with multiple acyl-CoA dehydrogenase (MADD) deficiency, increased expression of TFEB, LC3B-II and p62/SQSTM1 was detected in some atrophic muscle fibres, whereas in carnitine palmitoyltransferase II (CPT) deficiency, neither the formation of autophagosomes nor p62 protein aggregates was detected. Further studies are necessary to evaluate the contribution of autophagy to the pathogenesis of various β-FAO mitochondrial enzyme deficiencies.

The most thoroughly studied and clinically significant connection is between mitochondrial fatty acid oxidation enzyme deficiencies and mitophagy. The results of a recent study [85] demonstrated a previously unknown role of PINK1/Parkin-mediated mitophagy defects in β-FAO in cardiomyocytes of mice with a CPT2 mutation. In this case, suppression of the expression of the mitochondrial deubiquitinating enzyme (USP30) may promote ubiquitination of the outer mitochondrial membrane, which is necessary for the activation of mitophagy; this may compensate for mitochondrial damage in the context of impaired β-FAO. An earlier study [86] on fibroblasts from patients with MCAD deficiency showed that mutations in this enzyme lead to a significant reduction in the number of respiratory chain complexes (OXPHOS) and a decrease in oxygen consumption in mitochondria. The observed secondary mitochondrial dysfunction may be a key factor in the subsequent disruption of the mitophagy process.

The role of other selective autophagy processes, such as ER-phagy and lysophagy, in the development of β-FAO deficiencies is not yet entirely clear, but some studies show promising approaches. A 2020 study [87] showed that pharmacological inhibition of oxidative phosphorylation selectively suppresses ER-phagy without affecting overall macroautophagy. Since oxidative phosphorylation is disrupted in CPT2 deficiency, it can be assumed that ER-phagy may be an important link in the pathogenesis of β-FAO deficiencies. The link between lysophagy and β-FAO deficiencies is non-standard in nature. According to data from Vechalapu S.K. et al. (2025) [88], changes in lipid metabolism directly affect lysosomal function. This presumably means that chronic impairment of fatty acid oxidation and, consequently, the accumulation of lipid droplets in cells may damage lysosomes, forcing the cell to eliminate them via lysophagy.

Table 1 summarises approaches to modulating autophagy in mitochondrial diseases.

## 4. Conclusions

The data presented in this review demonstrate that autophagy and its selective forms play an important, multifaceted and often bidirectional role in the pathogenesis of mitochondrial diseases. Mitochondrial dysfunction caused by mutations in nuclear/mitochondrial DNA or a deficiency of mitochondrial enzymes not involved in oxidative phosphorylation inevitably affects the cell’s autophagic apparatus.

Mitophagy is the central and most extensively studied aspect in research into the role of autophagy in the pathogenesis of mitochondrial diseases. In mitochondrial syndromes (KSS, MERRF, MELAS) and fatty acid oxidation enzyme deficiencies (VLCAD, MCAD, CPT2), either enhanced but ineffective activation of mitophagy or a significant reduction in its activity is observed. The progression of mitochondrial disease over time leads to the depletion of cellular resources, resulting in the accumulation of damaged mitochondria, increased oxidative stress, and disruption of mitophagy processes, ultimately leading to non-selective cell death. Selective autophagy of other organelles is closely intertwined with mitochondrial function. Pexophagy is essential for the removal of peroxisomes during metabolic stress; however, its hyperactivation suppresses mitophagy. Reticulophagy (ER-phagy) and lipophagy directly regulate the transport of lipids into mitochondria and are sensitive to the cell’s energy status. Lysophagy, in turn, may be secondarily impaired due to ATP deficiency and changes in membrane lipid composition, creating a vicious circle. Aggrephagy is activated in response to proteotoxic stress caused by mitochondrial dysfunction, but its effectiveness is limited.

It should be noted that disruption of the autophagic pathway is not merely a consequence, but also a driver of disease progression. Many studies have demonstrated that restoring autophagy (through pharmacological activation or genetic engineering methods) improves mitochondrial function and cell survival. This suggests the possibility of modulating autophagy as a therapeutic strategy.

In conclusion, despite significant progress, many aspects of mitochondrial diseases remain poorly understood. In particular, the role of autophagy of cellular organelles, apart from mitophagy, has hardly been investigated. Studying these processes will enable the identification of new mechanisms of cellular pathogenesis of these diseases and potential approaches to pathogenetic therapy.

## Figures and Tables

**Table 1:** Therapeutic agents and approaches aimed at modulating the autophagic flux in mitochondrial diseases.

Mitochondrial Disorder	Disease Models	Therapeutic Treatment	Mechanism of Molecular Target Action	Clinical Status	Reference
Kerns–Seyer syndrome (KSS)	Different cell types from patients harbouring a single 4.9 kb mtDNA deletion	Amino acid supplements	Stimulation of mTOR and prevention of autophagy activation	Preclinical studies (in vitro)	[42]
MERRF syndrome	Primary cultured skin fibroblasts from patients with MERRF	Coenzyme Q10 (controversial)	It is assumed to prevent the translocation of Parkin to the mitochondria and to stimulate autophagy and mitophagy.	Preclinical studies (in vitro)	[45]
MELAS syndrome	Women with MELAS syndrome	Lamotrigine	No data	A single clinical case	[53]
	Fibroblasts from MELAS patients	Nitazoxanide	Activation of MNRR1 enhances mitophagy (via increased expression of PINK1) and mitochondrial biogenesis	Preclinical studies (in vitro)	[54]
	Fibroblasts from MELAS patients	Rapamycin	Inhibition of mTORC restored the autophagic flux and activated mitophagy, whilst also promoting the recovery of mitochondrial membrane potential and improving oxidative phosphorylation.	Preclinical studies (in vitro)	[55]
GNE myopathy	Fibroblasts from GNE myopathy patients	Metformin	Inhibition of the AMPK–mTOR signalling pathway, increased autophagy and increased cell viability	Preclinical studies (in vitro)	[58]
	C2C12 mouse myoblast model and isogenic human pluripotent stem cell-derived NMOs	Copanlisib	Inhibition of PI3K leads to the reactivation of the ULK1 pathway and the normalisation of autophagy.	Preclinical studies (in vitro)	[59]
	Skeletal muscle cell-based model system SKM-GNEHz	Hydroxyethylamine-based molecules—LTC-181 and LTC-1717	An increase in the LC3BII/I ratio, indicating the activation of autophagy.	Preclinical studies (in vitro)	[60]
Autophagic vacuolar myopathy	Lamp2 knockout (KO) mouse	Gene therapy (adenovirus-associated viral vectors)	Correction of a mutation in the LAMP2 gene by gene therapy	Preclinical studies (in vitro)	[66]
	Lamp2 knockout Danio rerio	Gene therapy (knockout mTOR)	Inhibition of mTOR by gene therapy reduces the adverse effects of impaired autophagy.	Preclinical studies (in vitro)	[68]
Myofibrillar myopathy	Danio rerio and human myoblasts	Metformin	Induction of the general autophagy process, removal of BAG3 protein aggregates, restoration of muscle fibre structure and normalisation of motor activity in fish with a bag3 gene knockout	Preclinical studies (in vitro)	[74]
Deficiency of mitochondrial fatty acid oxidation (VLCAD, MCAD, CPT)	FAO-deficient mice with CPT2 deletion	Gene therapy (knockout USP30)	Suppression of USP30 expression may contribute to the ubiquitination of the outer mitochondrial membrane and the activation of mitophagy.	Preclinical studies (in vitro)	[85]

## Data Availability

No new data were created or analysed in this study. Data sharing is not applicable to this article.

## Abbreviations

mTORC1	mechanistic target of rapamycin complex 1
AMPK	5′-prime-AMP-activated protein kinase
ULK1	Unc-51 Like Autophagy Activating Kinase
PI3KC	phosphoinositide 3-kinase complex
PI3P	phosphatidylinositol 3-phosphate
LC3B	microtubule-associated protein 1A/1B-light chain 3
LAMP	lysosomal-associated membrane protein
Ub	ubiquitin
BNIP3L/NIX	BCL2 Interacting Protein 3 Like
BNIP3	BCL2 Interacting Protein 3
FUNDC1	FUN14 domain containing 1
ROS	reactive oxygen species
PEX	peroxin protein
PMP	peroxisomal membrane protein
NBR1	neighbour of BRCA1 gene 1
NUFIP-1	nuclear fragile X mental retardation-interacting protein 1
ZNHIT3	zinc finger HIT domain-containing protein 3
ER	endoplasmic reticulum
LIR	LC3B-interacting region
AIM	ATG8-interacting motif
TRIM16	tripartite motif containing 16.
FBXO27	F-Box Protein 27
ELDR	endolysosomal damage response
TEX264	Testis Expressed 264
GABARAP	GABAA receptor-associated protein
LDs	lipid droplets
FXR	nuclear receptor farnesoid X receptor
PPARα	peroxisome proliferator-activated receptor alpha
CREB	transcriptional activator cAMP response element-binding protein
SOCE	store-operated calcium entry
KSS	Kearns–Sayre syndrome
CPEO	chronic progressive external ophthalmoplegia
mtDNA	mitochondrial DNA
NDP52	nuclear domain 10 protein 52
TAX1BP1	Tax1 binding protein 1
PERK	PRKR-like endoplasmic reticulum kinase
HSPA4	heat shock protein family A
DNAJC	DnaJ heat shock protein family
MERRF	myoclonic epilepsy with ragged-red fibres
MELAS	mitochondrial encephalomyopathy, lactic acidosis, and stroke-like episodes
iPSCs	induced pluripotent stem cells
RPE	retinal pigment epithelium
STAT3	signal transducer and activator of transcription 3
MNRR1	mitochondrial nuclear retrograde regulator 1
MFM	myofibrillar myopathy
CASA	chaperone-assisted selective autophagy
β-FAO	β-oxidation of fatty acids
VLCADD	very-long-chain acyl-CoA dehydrogenase deficiency
MCADD	medium-chain acyl-CoA dehydrogenase deficiency
CPT	carnitine Palmitoyltransferase
